# Comparative Analysis of Chloroplast Genomes within *Saxifraga* (Saxifragaceae) Takes Insights into Their Genomic Evolution and Adaption to the High-Elevation Environment

**DOI:** 10.3390/genes13091673

**Published:** 2022-09-19

**Authors:** Zhuyifu Chen, Xiaolei Yu, Yujiao Yang, Pei Wei, Wencai Zhang, Xinzhong Li, Chenlai Liu, Shuqi Zhao, Xiaoyan Li, Xing Liu

**Affiliations:** 1 State Key Laboratory of Hybrid Rice, College of Life Sciences, Wuhan University, Wuhan 430072, China; 2 Laboratory of Plant Systematics and Evolutionary Biology, College of Life Sciences, Wuhan University, Wuhan 430072, China; 3 Laboratory of Extreme Environmental Biological Resources and Adaptive Evolution, Research Center for Ecology, School of Sciences, Tibet University, Lhasa 850000, China; 4 Biology Experimental Teaching Center, School of Life Science, Wuhan University, Wuhan 430072, China

**Keywords:** chloroplast genome, *Saxifraga*, comparative analysis, hypervariable regions, high-elevation adaptation

## Abstract

*Saxifraga* species are widely distributed in alpine and arctic regions in the Northern hemisphere. Highly morphological diversity within this genus brings great difficulties for species identification, and their typical highland living properties make it interesting how they adapt to the extreme environment. Here, we newly generated the chloroplast (cp) genomes of two *Saxifraga* species and compared them with another five *Saxifraga* cp genomes to understand the characteristics of cp genomes and their potential roles in highland adaptation. The genome size, structure, gene content, GC content, and codon usage pattern were found to be highly similar. Cp genomes ranged from 146,549 bp to 151,066 bp in length, most of which comprised 130 predicted genes. Yet, due to the expansion of IR regions, the second copy of *rps19* in *Saxifraga stolonifera* was uniquely kept. Through sequence divergence analysis, we identified seven hypervariable regions and detected some signatures of regularity associated with genetic distance. We also identified 52 to 89 SSRs and some long repeats among seven *Saxifraga* species. Both ML and BI phylogenetic analyses confirmed that seven *Saxifraga* species formed a monophyletic clade in the Saxifragaceae family, and their intragenus relationship was also well supported. Additionally, the *ndhI* and *ycf1* genes were considered under positive selection in species inhabiting relatively high altitudes. Given the conditions of intense light and low CO_2_ concentration in the highland, the products of these two genes might participate in the adaptation to the extreme environment.

## 1. Introduction

A chloroplast is a unique, semiautonomous plant organelle responsible for providing nutrition and oxygen by converting light energy into chemical energy through oxygenic photosynthesis [1,2]. The cp genome is independent of the nuclear genome and contains plenty of genetic information. It is known for genetic stability characterized by the conserved circular structure, highly similar gene content, gene order, maternal inheritance, and lack of recombination [3]. For instance, the size of cp genomes for land plants ranges from 100 to 200 kb. The typical cp genomes share the quadripartite structure consisting of one large single copy region (LSC), one small single copy region (SSC), and two isometric inverted regions (IR), within which there are 120–130 genes for which products perform basic functions of various life activities, such as photosynthesis, transcription, translation, and so forth [2,4]. Predominantly maternal inheritance ensures the haplotype and identity of the cp genomes, while a large number of copies for the chloroplast in the cytoplasm also makes the cp genome more detectable. On the other hand, cp genomes were widely found to be variable in various genera or families, even in some closely related species [5,6]. It has been pointed out that some of these changes could reflect the genetic distance of species and be related to environmental adaptations [7]. Based on these traits, and with the improvements in next-generation sequencing technologies, cp genomes are more available and considered informative for intractable phylogenetic issues with high resolution [8]. Moreover, it is also usually applied for barcoding development and adaptive evolution [9].

*Saxifraga*, comprising more than 400 species, is the most species-abundant and classification-complex genus in the Saxifragaceae family [10,11,12]. As cold-adapted perennial herbs usually growing in alpine meadows and scrub, many *Saxifraga* species have been widely cultivated and studied for both ornamental and medical value. According to reports, extracts of several *Saxifraga* species possess antiparasitic, antioxidant, and antitumor properties [13,14,15]. *Saxifraga* is distributed mainly in the Northern hemisphere. It originated from North America and was further dispersed to Europe and the Qinghai–Tibet plateau (QTP) region. In China, since the rapid uplift and the forming of the extreme environment in the QTP, rapid radiation has occurred multiple times, and over 200 *Saxifraga* species, represented by Sect. Ciliatae and Sect. Porphyrion, have turned into a significant component of the QTP [16]. Some organisms have radiated out of the QTP. The difference in habitats also gives rise to multiple variations in morphology (e.g., cushion form, and lime-secreting hydathodes), karyotype, and reproduction, making it difficult to classify only by macroscopic features [17,18,19]. Therefore, it is necessary to seek more reliable and powerful molecular tools for better species identification and understanding of the phylogenetic relationship in the genus. Many efforts have been made for the phylogeny of *Saxifraga* [20,21]. Through large-scale sampling, Tkach et al. provided a relatively complete phylogeny of the *Saxifraga* genus [22]. Nevertheless, as multiple studies focused on the medical value and phylogeny of *Saxifraga* species, research discussing their adaptation to the unique alpine environment they inhabit is rare.

During diffusion to various alpine and arctic regions, the *Saxifraga* genus undergoes rapid diversification, which makes it the perfect material to investigate how the alpine area influences speciation and differentiation and to learn how these species adapt to the alpine environment [17]. Habitats with high altitudes usually have specific environmental characteristics, such as intense radiation, low temperature, and low CO_2_ concentration, which could leave some traces among environmentally adapted genes [23,24]. With topology reconstructed with protein-coding genes (PCGs), phylogenetic methods could be utilized to estimate the evolutionary rates of genes and detect the footprints of natural selection [25]. As the genome within an organelle that is essential to the life activities of green plants, the conservation and the irreplaceability of the cp genome provide a good opportunity to explore such footprints and their adaptative contribution. Many genome-wide adaptations to the high altitude have been reported [26,27,28]. However, there was only a minority of research working on the accelerating evolution among cp genomes induced by altitude adaptation [29,30]. For high-altitude species, genes in the *ndh* (*ndhA*, *ndhH*, and *ndhF*) and *ycf* (*ycf4*) families frequently showed a higher evolutionary rate when compared to low-altitude species. Considering the alpine environment that most *Saxifraga* species inhabit, it is feasible to study the selection their cp genomes go through during environmental adaptation.

In the present study, we would like to seek some clues from the cp genome that help to better understand genetic features and adaptive evolution in the *Saxifraga* genus. For the first time, we conducted a comprehensive cp genome characteristics and adaptation analysis of *Saxifraga*. First of all, cp genomes of two *Saxifraga* species, *Saxifraga sessiliflora* and *Saxifraga saginoides*, were newly sequenced. Together with four available cp genomes (*Saxifraga sinomontana*, *Saxifraga umbellulata var. pectinata, Saxifraga umbellulata var. umbellulata,* and *S. stolonifera*) in GenBank and one newly assembled cp genome (*Saxifraga granulata*) with DNA data from NCBI, several comparative analyses were performed to investigate the genetic features of the cp genome within *Saxifraga*, including the genome size, structure, gene content, GC content, IR boundary, nucleotide diversity, codon usage, and SSR distribution. Then, another forty-three cp genomes in the Saxifragales order were combined to reconstruct a partial topology of the Saxifragaceae family and investigate the phylogenetic relationships of seven *Saxifraga* species within the Saxifragaceae family. Moreover, some genes and sites accelerating evolution that were probably induced by the harsh environment were investigated at the genus level. Above, in this study, we aimed to (1) take a glimpse of the characteristics and evolution of the cp genome within the *Saxifraga* genus and (2) figure out whether natural selection has exerted an influence on the cp genome of *Saxifraga* to make it adapt to the extreme high-elevation environment.

## 2. Materials and Methods

### 2.1. Plant Material Sampling and DNA Sequencing

Seven *Saxifraga* species were included in this study, two of which were newly collected from the QTP, China, at an altitude of 4898 m in 2021 (Table S8). Both samples were identified by Professor Xing Liu from Wuhan University in China, and the voucher specimens were deposited in the herbarium of Wuhan University. The young, fresh, and healthy leaves were collected and instantly frozen in liquid nitrogen and later restored at −80 °C. Total genomic DNA was extracted by the modified CTAB method. After the integrity and concentration test and library construction, genomic DNA was fragmented and ligated with adapters. The genomic DNA was sequenced on Illumina Novaseq 6000 platform through further amplification and purification. Raw data were deposited in the NCBI Sequence Read Archive (SRA) under accession numbers SRR20740825 and SRR20740826, respectively.

### 2.2. De Novo Assembly and Annotation

Raw data were filtered with fastp software by removing adapter sequences and low-quality reads [31]. GetOrganelle was used for de novo assembly of the cp genome for *S. saginoides* and *S. sessiliflora* [32]. Subsequently, the assembled contig was corrected by pilon and confirmed with short-read mapping to contig through bowtie2 [33,34]. The result was visualized on geneious 8.0.4. Through alignment, the whole contig was fully covered by clean data. Then, the well-assembled sequence was annotated by Geseq [35]. tRNA genes were identified using tRNAscanSE with the default setting [36]. After aligning with a set of reference cp genomes, initial annotations were manually checked and adjusted to validate each gene’s initial codon, terminal codon, and intron position. The newly obtained cp genomes were deposited in GenBank under accession numbers ON458148 and ON458149. These two cp genomes are also accessible in the figshare database [37].

### 2.3. Sequence Divergence Analysis and Visualization

To visualize the position, transcriptional direction of genes, and the structure feature of each cp genome, OrganellarGenomeDRAW was used to generate the physical maps of *S. saginoides* and *S. sessiliflora* [38]. Variation region of cp genome among *Saxifraga* genus was identified and visualized utilizing mVISTA program with a LAGAN mode [39,40]. The online tool IRscope was used for further exhibiting gene distribution at the boundaries of SSC, LSC, IRa, and IRb [41]. Seven cp genomes were aligned together. The nucleotide polymorphism (Pi) among cp genomes was calculated and visualized using DnaSP software, with a window length of 600 bp and a step size of 200 bp [42].

### 2.4. Calculation of Codon Usage

Mega X was applied to calculate the relative synonymous codon usage (RSCU) of all the PCGs [43]. RSCU represented the preference for codon usage. RSCU of one codon greater than 1 means it was preferred when coding the same amino acid. GC3s and ENC of each PCG among cp genomes were calculated using CodonW v1.4.4 (JF Peden, Nottingham, UK), and then both values were compared, with the standard curve calculated and visualized using R script. ENC represents the effective number of codons, and it is one of the most informative parameters to estimate the degree of imbalanced usage for synonymous codons [44]. Greatly preferred synonymous codons hold lower ENC values. GC3 means the GC content of synonymous codons at the third position. The standard curve in the ENC-GC3 plot represents the fit of the formula ENC -GC3 content. If the calculated ENC value of a gene was approximate to the standard curve, the observed codon bias was due primarily to nucleotide composition difference at the third codon position, which was mainly influenced by mutation [45]. On the contrary, it was much influenced by natural selection and other factors. Some genes, such as *psbL* and *psbM*, were not calculated because their length was overly short [46].

### 2.5. Identification of Repeat Sequences in Organelle Genomes

Simple sequence repeats (SSRs) of cp genomes were identified using MISA, and the parameters were as follows: 10, 5, 4, 3, 3, and 3 for mono-, di-, tri-, tetra-, penta-, and hexanucleotides, respectively [47]. The size and location of long repeats were determined by REPuter, with a minimum repeat size of 30 bp and a hamming distance of 3 [48].

### 2.6. Phylogenetic Analysis

Fifty species (forty-seven species among Saxifragaceae and three outgroups) were selected to reconstruct a phylogenetic tree. After manually checking and adjusting, 79 shared PCGs were extracted, aligned, and concatenated to a matrix using PhyloSuite [49]. Then, the phylogenetic tree was reconstructed by setting Myriophyllum spicatum, Ribes nevadense, and Ribes roezlii as outgroups. Maximum likelihood (ML) analyses were made using IQ-TREE with the GTR+R3+F model automatically selected by ModelFinder for 5000 ultrafast bootstraps [50]. Then, Bayesian inference (BI) phylogenies were carried out using MrBayes 3.2.6 with the GTR+I+G+F model (2 parallel runs, 2,000,000 generations), in which the original 25% of sampled data were thrown away as burn-in [51]. Two constructed trees were visualized in the Interactive Tree Of Life [52].

### 2.7. Selective Analysis

We calculated the Ka/Ks (Ka for nonsynonymous substitution ratio and Ks for synonymous substitution ratio) ratio among seven *Saxifraga* species. Seventy-nine shared PCGs were extracted separately by PhyloSuite and simultaneously translated to the amino acid sequence. ParaAT was used to prepare intermediate files automatically and calculate Ka/Ks value by calling KaKs_calculator 2.0 [53,54]. Some dispersed values greater than 45 or some rows (*atpH*, *petN*, *psaJ*, *psbF*, *psbI*, *psbJ*, *psbK*, *psbL,* and *psbM*) and columns (*S. saginoides*-*S. sinomontana*) with too much Na were discarded [55]. This could be caused by the exceedingly low synonymous substitution ratio. Ka/Ks ratio >1 indicated that the gene pair was considered under accelerated selection, while Ka/Ks ratio <1 was regarded as under purifying selection.

Branch-site model in the codeml program of PAML was applied to estimate the selective pressure caused by environmental adaptation among *Saxifraga* [25]. Selective pressure was quantified by the ratio(ω) of the nonsynonymous substitution rate(dN) to the synonymous substitution rate (dS). Through the likelihood ratio test (LRT), the alternative model (“model = 2, NSsites = 2, omega = 0.5|1.5, fix_omega = 0”) was compared with the null model (“model = 2, NSsites = 2, omega = 1, fix_omega = 1”). The P-value of LRT was acquired by the Chi-squared test. Moreover, the BEB method was implemented to test and select amino acid sites that were potentially under positive selection. A gene with a *p*-value < 0.05 and ω > 1 was supposed to be under positive selection, and an amino acid site with posterior probabilities > 0.95 was considered under significantly positive selection.

## 3. Results and Discussion

### 3.1. Characteristics of the CP Genome for Saxifraga Species

**Figure 1:**
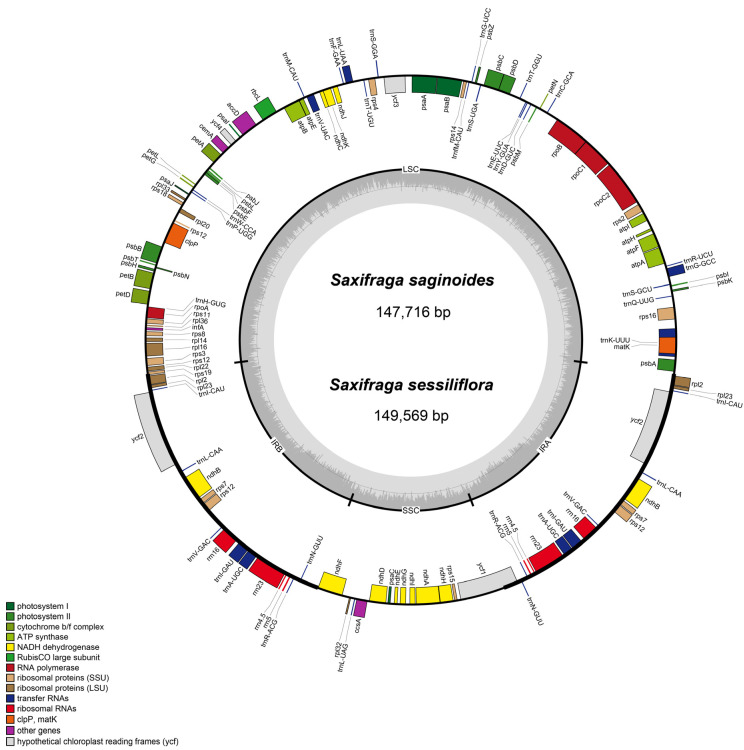
cp genome map of two newly sequenced *Saxifraga* species, *S. saginoides* and *S. sessiliflora*. The outer circle shows the transcription direction; genes outside are transcribed clockwise while genes inside are transcribed counterclockwise. LSC/SSC/IR regions are exhibited in the inner circle. Genes of different functions are presented in different colors.

After filtering, two newly sequenced species generated more than three gigabases (Gb) of clean data. Both data were assembled to high-quality contigs without any gap when mapped with clean short reads. Contigs were cyclized, well annotated, and manually checked with some other cp genomes within the Saxifragaceae family (Figure 1; Table 1). By and large, cp genomes among seven *Saxifraga* species exhibited similar signatures (Table 2). All the seven cp genomes exhibited typical quadripartite structures, which consisted of two isometric IR regions (IRb/IRa, ranging from 25,412 to 25,651 bp), one LSC region (79,310–82,738 bp), and one SSC region (16,390–17,504 bp). *S. umbellulata var. pectinata* had the smallest cp genome size (146,549 bp), while *S. stolonifera* had the largest (151,066 bp). The overall GC (guanine–cytosine) content was nearly identical (37.8–38.1%), and the GC content of two IR regions (42.8–43%) was higher than that of the LSC (35.8–36.2%) and SSC (31.9–32.4%) regions. It was apparent that cp genomes showed AT preference, and such a preference was most prominent in the SSC region. Additionally, all the cp genomes also had a highly similar gene content. Most of them comprised 79 unique PCGs, 30 unique tRNA genes, and 4 unique rRNA genes, among which 6 PCGs, 7 tRNA genes, and 4 rRNA genes located at IR regions were duplicated (Table 1 and Table 2). *S. stolonifera* contained two copies of the *rps19* gene, while the second copy of *rps19* in the other six cp genomes was under pseudogenization. For all cp genomes, 17 PCGs and tRNA genes were detected to contain introns, and 3 PCGs, *rps12*, *clpP*, and *ycf3*, comprised two introns (Table 1). Compared to cp genomes from other genera of Saxifragaceae, there were no significant differences in genome size, GC content, gene content, and gene order, which was congruent with other higher plants [56,57].

### 3.2. IR Boundary Analysis

**Figure 2:**
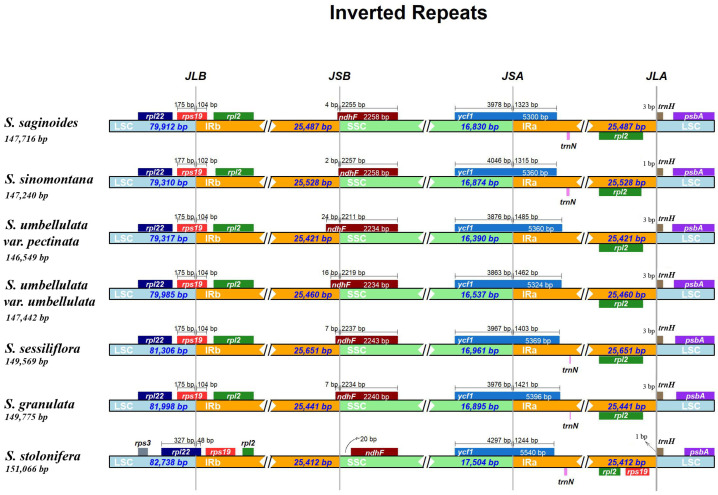
A plot displaying the boundaries of the LSC, SSC, and IR regions and genes around the junction sites for each *Saxifraga* cp genome. JLB, JSB, JSA, and JLA denote the junction sites of LSC and IRb, IRb and SSC, SSC and IRa, and IRa and LSC, respectively.

The IR/SC boundary shift was widely reported as a general evolutionary phenomenon, which reflected the expansion and contraction of the cp genome and made genes near borders pseudogenization [58,59,60]. We investigated the border of IR, LSC, and SSC regions among *Saxifraga* species, and some variations are displayed in Figure 2 Through comparison, the boundaries showed no significant differences. In most *Saxifraga* species, the *ndhF* gene was mainly on the SSC region, and its right end had a little on the IRb region; in the *S. stolonifera*, *ndhF* was sited on the SSC region, without any part on the IRb. The *ycf1* gene took both sides of the SSC and Ira region, and its length ranged from 5300 to 5540. Due to the special location, another copy of *ycf1*, located at the boundary of the IRb and SSC region, became a truncated pseudogene. The *rps19* gene was largely located at the junction of LSC and IRb and, similar to *ycf1*, most of the second copy at the boundary of the IRa and LSC region was also under pseudogenization. In contrast, the *rps19* genes of *S. stolonifera* were totally embedded in the IR regions so that pseudogenization of the second copy was avoided. The result also reflected from the side the expansion event experienced on the IR boundary of the *S. stolonifera*.

### 3.3. Genomic Sequence Divergence

Similar to SSR markers, barcoding is a useful molecular tool for identifying organisms [61,62]. For a long time, *rbcL*, *matK*, and *trnH-psbA* in the cp genome, and ITS in nuclear sequence, constituted universal markers due to their relatively high specificity and amplification efficiency [63]. Nevertheless, they were not specific enough to distinguish closely related species in many cases. Therefore, it is necessary to search specific markers for precise identification.

**Figure 3:**
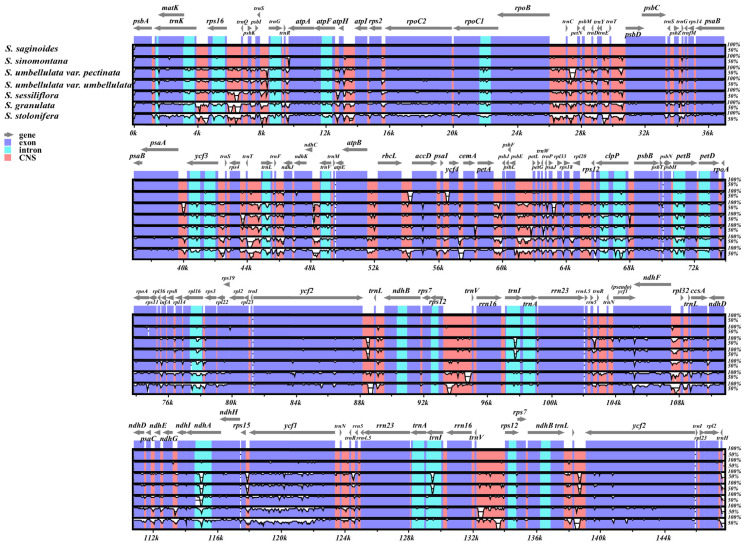
The alignment and comparative analysis of the whole cp genome for seven *Saxifraga* species. Among them, *S. saginoides* was set as reference. The horizontal axis represents the coordinates of cp genomes in the alignment result. Exons, introns, and conserved noncoding sequences (CNSs) were marked as different colors.

First, mVISTA was used to visualize overall changes in the cp genomes among *Saxifraga* species. The complete cp genome of *S. saginoides* was used as a reference to compare with the other six cp genomes (Figure 3). As expected, most PCGs showed high consistency. Several obvious variations were shown in noncoding regions, such as intergenic regions such as *trnK-rps16*, *rps16-trnQ,* and the intron of the *ndhA*. Remarkably, *S. stolonifera* and *S. granulata* exhibited more and larger variance than other *Saxifraga* species, which was consistent with the phylogenetic relationship that these two species belonged to different sections from the other five species inferred by the previous study [22].

**Figure 4:**
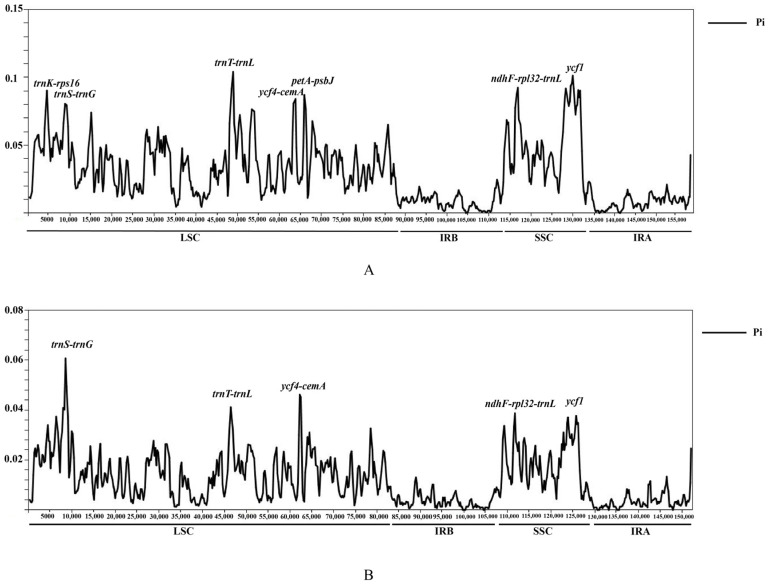
Comparative analysis of the nucleotide polymorphism (Pi) values among the cp genomes of *Saxifraga*. (**A**) Pi values calculated as all the seven *Saxifraga* species were taken into account. (**B**) Pi values calculated as five species in Sect. Ciliatae was selected separately.

The Pi value was then calculated among these *Saxifraga* cp genomes to confirm the visual result acquired from mVISTA and further detect the hypervariable regions. From Figure 4, both results suggested that IR regions were much more conserved than the LSC and SSC regions. When conducting analyses among all seven species, the Pi value ranged from 0 to 0.1 among the whole cp genome (Figure 4A; Table S1). Six intergenic regions (*trnK-rps16*, *trnS-trnG*, *trnT-trnL*, *ycf4-cemA*, *petA-psbJ*, and *ndhF-rpl32-trnL*) and part of gene *ycf1* presented higher variability (0.07–0.1) than other regions, which was in accordance with what was exhibited in the mVISTA analysis. *trnT-trnL* was the most volatile region with a Pi value of 0.1. Additionally, Pi of five Sect. Ciliatae cp genomes (*S. saginoides*, *S. sessiliflora*, *S. sinomontana*, *S. umbellulata var. pectinata*, and *S. umbellulata var. umbellulata*) were calculated, and the value (0–0.06) was much lower than the Pi among seven *Saxifraga* species (Figure 4B; Table S1) [22]. Combining the twice-time Pi calculation with the mVISTA result shown above, we found that the whole cp genome exhibited characteristics of regularity associated with the phylogenetic relationship of seven *Saxifraga* species. Notably, once the non-Sect. Ciliatae species, *S. stolonifera* and *S. granulata*, were included, the regions with larger variation have changed. This indicated that different sections of a genus might provide some specific variations and searching hypervariable loci at the section level might offer the possibility for more precise species identification.

### 3.4. Condon Usage Analysis

**Figure 5:**
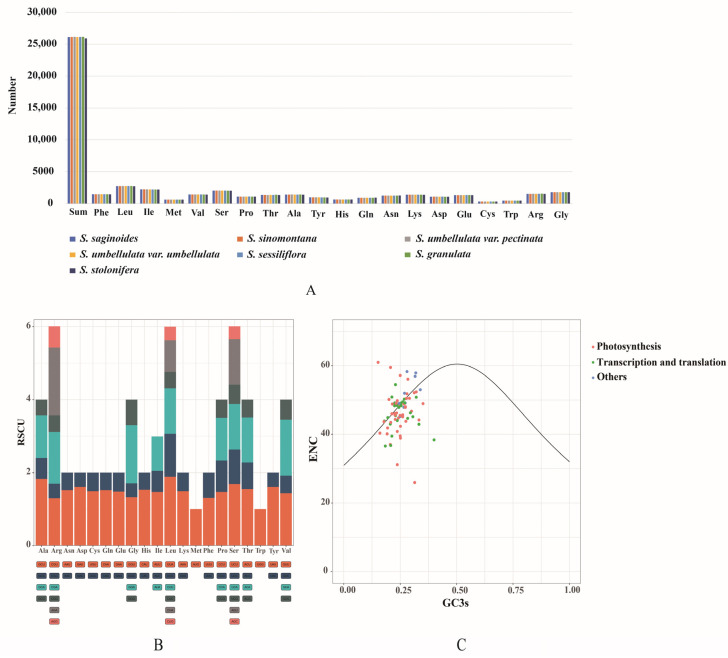
Usage preference of amino acids (AAs) and codons for PCGs. (**A**) AA usage of all the PCGs in each *Saxifraga* species. (**B**) RSCU for every AA in *S. saginoides*. For each amino acid, a color represents a unique codon. (**C**) ENC-GC3 plot for *S. saginoides*; every gene was displayed as a dot, and different colors mean genes in distinct functional groups.

Codon usage bias is vital for the reflection of the cp genome evolution. Generally, mutation, natural selection, phylogenetic relationship, and other factors may lead to diverse codon usage preferences [64,65]. In this study, codon usage bias and relative synonymous codon usage (RSCU) of the shared PCGs were analyzed among seven *Saxifraga* species. The *Saxifraga* genus exhibited highly similar codon usage preference and amino acid frequency (Figure 5A,B and Figure S1; Table S2). A total of 25,913 to 26,154 codons were identified in shared PCGs. Leucine (10.5–10.6%), Isoleucine (8.4–8.5%), and serine (7.7–7.8%) were widely used, while Cysteine (1.2%), tryptophan (1.7–1.8%), and methionine (2.3–2.4%) were less used. Most amino acids were coded with more than one synonymous codon because of codon degeneracy, such as Leucine with six codons and Isoleucine with four codons. However, only tryptophan and methionine had no alternative codon [66]. Similar to the other advanced plant, for those that applied more than one codon to code, the third nucleotide of the codon was frequently occupied by A/T instead of C/G [67].

Another analysis for ENC and GC3 was conducted on each PCG. The result suggested that PCGs of *Saxifraga* species shared consistent codon bias patterns (Figure 5C and Figure S2; Table S3). The calculated ENC of most genes appeared to range from 30 to 60. The majority of PCGswere in the vicinity of the expected ENC, suggesting that random mutation dominated these genes. A few photosynthesis-related genes and translation-related ribosomal proteins are distributed far below the standard curve, suggesting that natural selection or other factors might take effect.

### 3.5. Repeat Sequence Analysis

**Figure 6:**
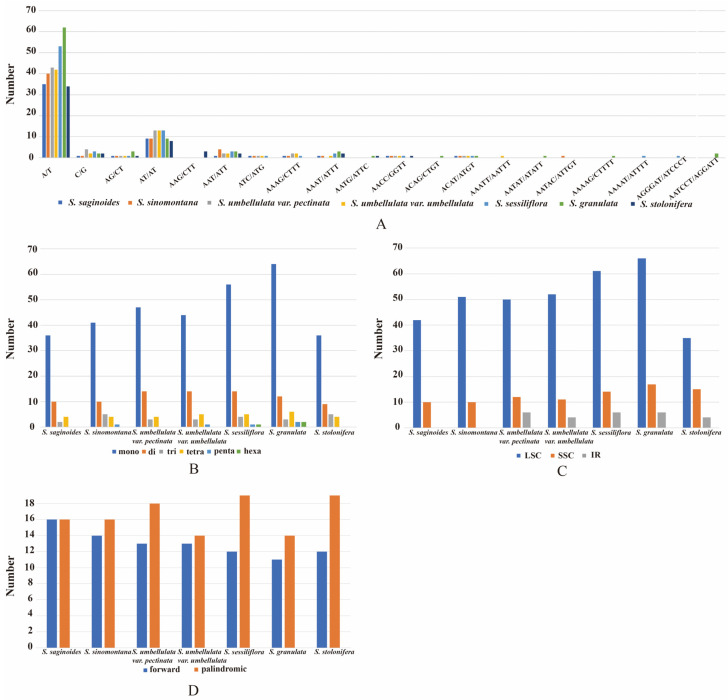
Repeats analysis among cp genomes of *Saxifraga*. (**A**) Distribution of all repeat units for SSRs in each species. (**B**) The number of different types for SSRs in each species. (**C**) Distribution of SSRs, respectively, in LSC, SSC, and IR regions. (**D**) The number of different types for long repeats.

SSRs have been described as a robust tool for species identification, population genetics, and phylogenetic studies [68,69,70,71]. Fifty-two to eighty-nine SSRs were identified in seven *Saxifraga* species (Figure 6B; Table S4). *S. granulata* contained the largest number of SSRs. Among these repeats, the mononucleotide SSRs were the most abundant (36–64) and mainly constituted by A/T. Some regular repeats, such as A/T/C/G, AG/CT/AT, and AAT, were shared by all cp genomes, whereas other repeat units with more than four nucleotides, such as AATG/ATTC, AATAT/ATATT, and AATCCT/AGGATT, were much more distinct in the specific cp genome (Figure 6A). For all cp genomes, LSC consisted of the greatest number of SSRs and two IRs included the least, which was congruent with the regularity of Pi analysis mentioned earlier (Figure 6C). Repeats longer than 30 bp were also found in seven cp genomes. Only forward and palindromic repeats appeared in all *Saxifraga* cp genomes (Figure 6D; Table S5). Some large, dispersed repeats were thought to be associated with the rearrangement and played an important role in genome evolution [72,73,74]. In summary, these repeat sequences will be helpful for subsequent population genetics studies.

### 3.6. Phylogenetic Analysis

**Figure 7:**
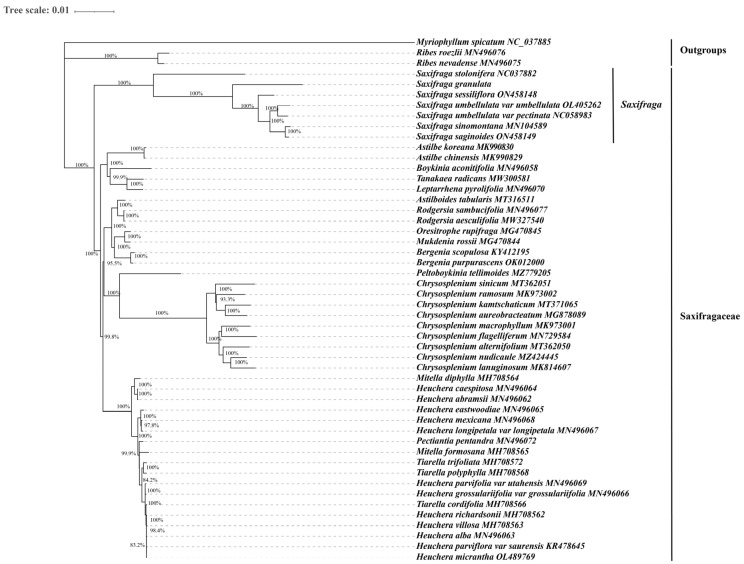
Maximum likelihood (ML) phylogenetic tree of 47 species in the Saxifragaceae family, reconstructed with 79 PCGs. Three non-Saxifragaceae species, Myriophyllum spicatum, Ribes nevadense, and Ribes roezlii, were set as outgroups.

To investigate the phylogenetic relationship of seven *Saxifraga* species in this study and the phylogenetic position of the *Saxifraga* genus in the Saxifragaceae family, 79 unique genes shared by the cp genomes of 50 species were applied to reconstruct the phylogenetic tree by both the ML and BI methods. All 50 species belonged to Saxifragales, within which 47 species were in the Saxifragaceae family, and 3 non-Saxifragaceae species, Myriophyllum spicatum (Haloragaceae), Ribes nevadense (Grossulariaceae), and Ribes roezlii, were set as outgroups. Both methods generated nearly identical topology, and all nodes were well supported with exceedingly high ML bootstrap and Bayesian posterior probability (Figure 7 and Figure S3). At the intrageneric level, *S. stolonifera*, *S. granulata*, and the rest of the *Saxifraga* species were divided into three clades. This result shared the same opinion with the previous phylogenetic study that *S. stolonifera* and *S. granulate*, respectively, belong to Sect. Irregulares and Sect. Saxifraga, and the rest of the species are located at Sect. Ciliatae [22]. In addition, *Saxifraga* species formed a monophyletic clade and were separate from all other species and groups in the Saxifragaceae family, which confirmed another previous study at the family level [75]. This suggested that the whole cp genomes could provide a high resolution of genetic information for an accurate phylogenetic relationship. Some researchers also pointed out that the cp genome could be utilized to accurately parse monophyletic phylogeny, while more complex phylogeny events, such as hybridization and plastid capture, needed nuclear sequence information to be united [76].

### 3.7. Selection and Adaption Analysis

**Figure 8:**
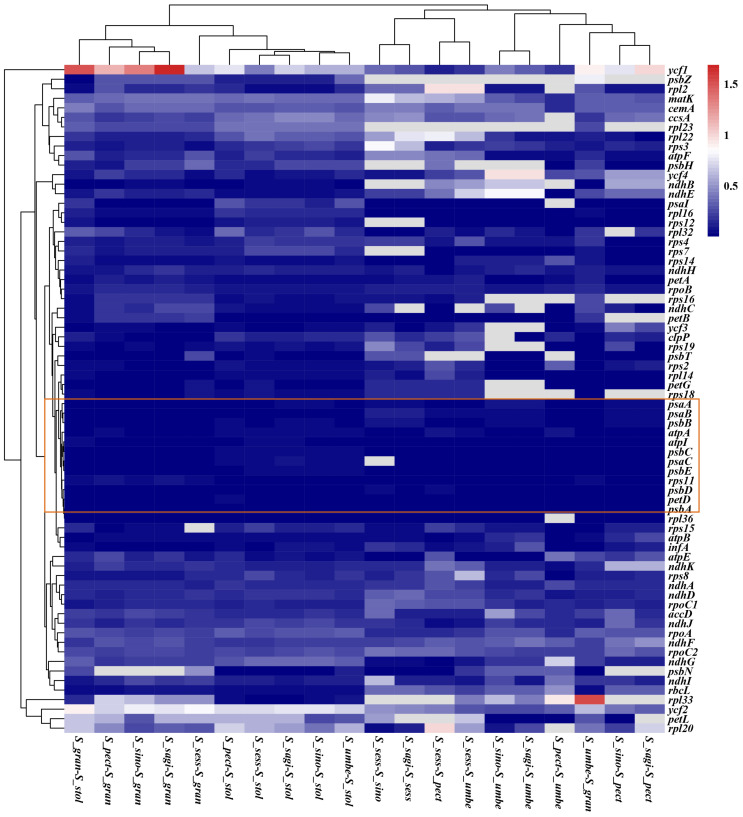
Heatmap representing pairwise Ka/Ks ratios of PCGs among *Saxifraga* species. The color bias toward red indicates that there is a higher Ka/Ks ratio between genes.

First of all, we calculated the Ka/Ks ratio of 79 PCGs between any two *Saxifraga* species. Most genes were under purifying selection (ratio lower than 1), and the majority ranged from 0.001 to 0.3, indicating that most PCGs were extremely conserved among *Saxifraga* cp genomes at the amino acid level (Table S6). According to the heatmap, the most conserved genes framed in the red line were suggested to be photosynthesis-related genes (Figure 8). There was only a minority of genes such as *ycf1* genes in the comparison of *S. umbellulata var. pectinata* and *S. granulata*, *S. sinomontana* and *S. granulata*, *S. granulata* and *S. stolonifera*, and *S. saginoides* and *S. granulata*, and *rpl33* in the comparison of *S. umbellulata var. umbellulata* and *S. granulata* being under accelerated selection (ratio higher than 1).

Seventy-nine unique PCGs were used to detect the natural selection pressure among *Saxifraga* species. Compared to other *Saxifraga* species mainly distributed in high-altitude areas, *S. stolonifera* inhabited regions with relatively lower altitudes, which means a moderate climate and weaker light radiation [10]. Therefore, it was worth investigating whether species inhabiting higher elevations with harsh environments underwent adaptive evolution. Selection pressure was estimated with the branch-site model by setting five species (*S. sinomontana*, *S. umbellulata var. pectinata*, *S. umbellulata var. umbellulata*, *S. sessiliflora*, and *S. saginoides*) living in the relatively high-altitude niche as the foreground. Genes *ndhI* and *ycf1* were found under positive selection (Table S7). As mentioned earlier, the ndh and ycf families were often involved in the adaptation to highland environments, which now has also been confirmed in the *saxifraga* species [29,30]. *ndhI* coded one of the components of the NAD(P)H dehydrogenase (NDH) complex. The NDH complex acted on the photosystem I cyclic electron transfer [77]. It was crucial for carbon accumulation and plant responses to environmental stresses, such as oxidation and fluctuations of light and temperature [78,79]. Given the natural selection of intense light and low air density on the QTP, the alteration of *ndhI*’s molecular sequence might participate in the adaptive response of *Saxifraga* species to environmental stress. The ycf1 gene encoded the protein TIC214 that acted as a protein-conducting channel at the inner envelope for importing protein precursors into chloroplasts [80,81]. As one of the longest genes in the cp genome, ycf1 presented substantial variation among various cp genomes, and whether it was essential was controversial [82]. Therefore, the reason why the *ycf1* gene was positively selected was unclear. Additionally, a few positively selected sites were also found in another 26 genes, which might also go through faster evolution due to the greatly stressful environment. 31T in the *psbT* gene and 159L in the *ndhI* gene were significantly proved to be positively selected by BEB posterior probability greater than 0.95.

## 4. Conclusions

In this study, seven *Saxifraga* species were selected for comparative, phylogenetic, and adaptive analysis of *Saxifraga* cp genomes. Overall, it was revealed that all the *Saxifraga* species shared similar cp genome size, structure, GC content, gene content, and genome components, and no rearrangements occurred in gene order, either. In the sequence divergence analysis, we detected seven hypervariable regions (*trnK-rps16*, *trnS-trnG*, *trnT-trnL*, *ycf4-cemA*, *petA-psbJ*, *ndhF-rpl32-trnL*, and gene *ycf1*) in seven *Saxifraga* species, which provided favorable materials for the further precise identification of *Saxifraga* species. In this part, IR regions were also found to be more conserved than the LSC and SSC regions. The phylogenetic tree reconstructed with the PCGs of cp genomes provided further evidence that the seven *Saxifraga* species in this study belong to three different sections. Meanwhile, *Saxifraga* species formed a monophyletic group in the Saxifragaceae family. The phylogenetic relationship was also reflected in several other features of the cp genome, such as the variation of the whole cp genome exhibited in the mVISTA analysis, and the Pi value calculated among different sections. Regarding altitude adaptation, at the branch and site level, for species inhabiting relatively high altitudes, the *ndhI* and *ycf1* genes were suggested to be under positive selection, which implied the adaptive contribution of the two genes to the extreme environment. The findings above provided some knowledge of the conservation and divergence of the *Saxifraga* cp genomes and laid the groundwork for precise species identification. Certainly, sampling on a larger scale is needed to learn more about the evolutionary features of *Saxifraga* cp genomes and the rules of environmental adaptation.

## Figures and Tables

**Table 1:** Gene annotation of the *S. saginoides* and *S. sessiliflora* chloroplast genome.

Category	Group	Genes
Photosynthesis related genes	Rubisco	*rbcL*
	Photosystem I	*psaA*, *psaB*, *psaC*, *psaI*, *psaJ*
	Photosystem II	*psbA*, *psbB*, *psbT*, *psbK*, *psbI*, *psbH*, *psbM*, *psbN*, *psbD*, *psbC*, *psbZ*, *psbJ*, *psbL*, *psbE*, *psbF*
	ATP synthase	*atpA*, *atpB*, *atpE*, *atpF ^a^*, *atpH*, *atpI*
	Cytochrome b/f complex	*petA*, *petB ^a^*, *petD*, *petN*, *petL*, *petG*
	Cytochrome C synthesis	*ccsA*
	NADPH dehydrogenase	*NdhA ^a^*, *ndhB ^a,c^* (×2), *ndhC*, *ndhD*, *ndhE*, *ndhF*, *ndhH*, *ndhG*, *ndhJ*, *ndhK*, *ndhI*
Transcription and translation related genes	Transcription	*rpoA*, *rpoB*, *rpoC2*, *rpoC1 ^a^*
	Ribosomal proteins	*rps2*, *rps3*, *rps4*, *rps7 ^c^* (×2), *rps8*, *rps11*, *rps12 ^b,c^* (×2), *rps14*, *rps15*, *rps16 ^a^*, *rps18*, *rps19*, *rpl2 ^a,c^* (×2), *rpl14*, *rpl16 ^a^*, *rpl20*, *rpl22*, *rpl23 ^c^* (×2), *rpl32*, *rpl33*, *rpl36*
	Translation initiation factor	*infA*
RNA genes	Ribosomal RNA	*rrn16S ^c^* (×2), *rrn23S ^c^* (×2), *rrn4.5 ^c^* (×2), *rrn5 ^c^* (×2)
	Transfer RNA	*trnH-GUG*, *trnK-UUU ^a^*, *trnQ-UUG*, *trnS-GCU*, *trnS-UGA*, *trnS-GGA*, *trnG-GCC ^a^*, *trnR-UCU*, *trnR-ACG ^c^* (×2), *trnC-GCA*, *trnD-GUC*, *trnY-GUA*, *trnE-UUC*, *trnT-UGU*, *trnG-UCC*, *trnfM-CAU*, *trnL-CAA ^c^* (×2), *trnL-UAA ^a^*, *trnL-UAG*, *trnF-GAA*, *trnV-GAC ^c^* (×2), *trnV-UAC ^a^*, *trnM-CAU*, *trnT-GGU*, *trnW-CCA*, *trnP-UGG*, *trnI-CAU ^c^* (×2), *trnI-GAU ^a,c^* (×2), *trnA-UGC ^a,c^* (×2), *trnN-GUU ^c^* (×2)
Other genes	RNA processing	*matK*
	Carbon metabolism	*cemA*
	Fatty acid synthesis	*accD*
	Proteolysis	*ClpP ^b^*
	Conserved ORFs	*ycf1*, *ycf2 ^c^* (×2), *ycf3 ^b^*, *ycf4*

*^a^* genes with one intron, *^b^* genes with two introns, *^c^* two gene copies in IRs.

**Table 2:** Summary statistics of chloroplast genomes for *Saxifraga* species.

Genome Feature	*S. saginoides*	*S. sessiliflora*	*S. sinomontana*	*S. umbellulata var. pectinata*	*S. umbellulata var. umbellulata*	*S. granulata*	*S. stolonifera*
Genome size (bp)	147,716	149,569	147,240	146,549	147,442	149,775	151,066
LSC size (bp)	79,912	81,306	79,310	79,317	79,985	81,998	82,738
SSC size (bp)	16,830	16,961	16,874	16,390	16,537	16,895	17,504
IR size (bp)	25,487	25,651	25,528	25,421	25,460	25,441	25,412
Number of genes	130	130	130	130	130	130	131
Protein genes	85	85	85	85	85	85	86
tRNA genes	37	37	37	37	37	37	37
rRNA genes	8	8	8	8	8	8	8
Duplicated genes in IRs	17	17	17	17	17	17	18
GC content (%)	38%	37.9%	38%	38.1%	38.1%	37.8%	37.8%
GC content in LSC (%)	36.2%	36%	36.2%	36.2%	36.2%	35.8%	35.9%
GC content in SSC (%)	32.1%	32%	32%	32.4%	32.4%	31.9%	32.2%
GC content in IRs (%)	42.9%	42.8%	42.9%	42.8%	42.8%	42.9%	43%

## Data Availability

The chloroplast sequences of *S. sessiliflora* and *S. saginoides* have been uploaded to GenBank and the accession numbers are ON458148 and ON458149. The genbank format files of two cp genomes are also available in the figshare database (DOI: https://doi.org/10.6084/m9.figshare.20488218.v2) (accessed on 19 August 2022).

## Supplementary Materials

Supplementary materials can be found at https://www.mdpi.com/article/10.3390/genes13091673/s1. Figure S1. RSCU for other six *Saxifraga* species. (A) *S. sinomontana*. (B) *S. umbellulata var. pectinata*. (C) *S. umbellulata var. umbellulata*. (D) *S. sessiliflora*. (E) *S. granulata*. (F) *S. stolonifera*.; Figure S2. ENC-GC3 plot for other six *Saxifraga* species. (A) *S. sinomontana*. (B) *S. umbellulata var. pectinata*. (C) *S. umbellulata var. umbellulata*. (D) *S. sessiliflora*. (E) *S. granulata*. (F) *S. stolonifera*; Figure S3. Bayesian inference (BI) phylogenetic tree of 47 species in Saxifragaceae family reconstructed with 79 protein-coding genes. Three non-Saxifragaceae species were set as outgroups; Supplementary table file: Tables S1–S7.
