# Supplementary figures and images for: Comparative Analysis of Chloroplast Genomes within Saxifraga (Saxifragaceae) Takes Insights into Their Genomic Evolution and Adaption to the High-Elevation Environment
Source: Genes (Basel). 2022 Sep 19;13(9):1673. doi: 10.3390/genes13091673 (PMC9498722; doi:10.3390/genes13091673)

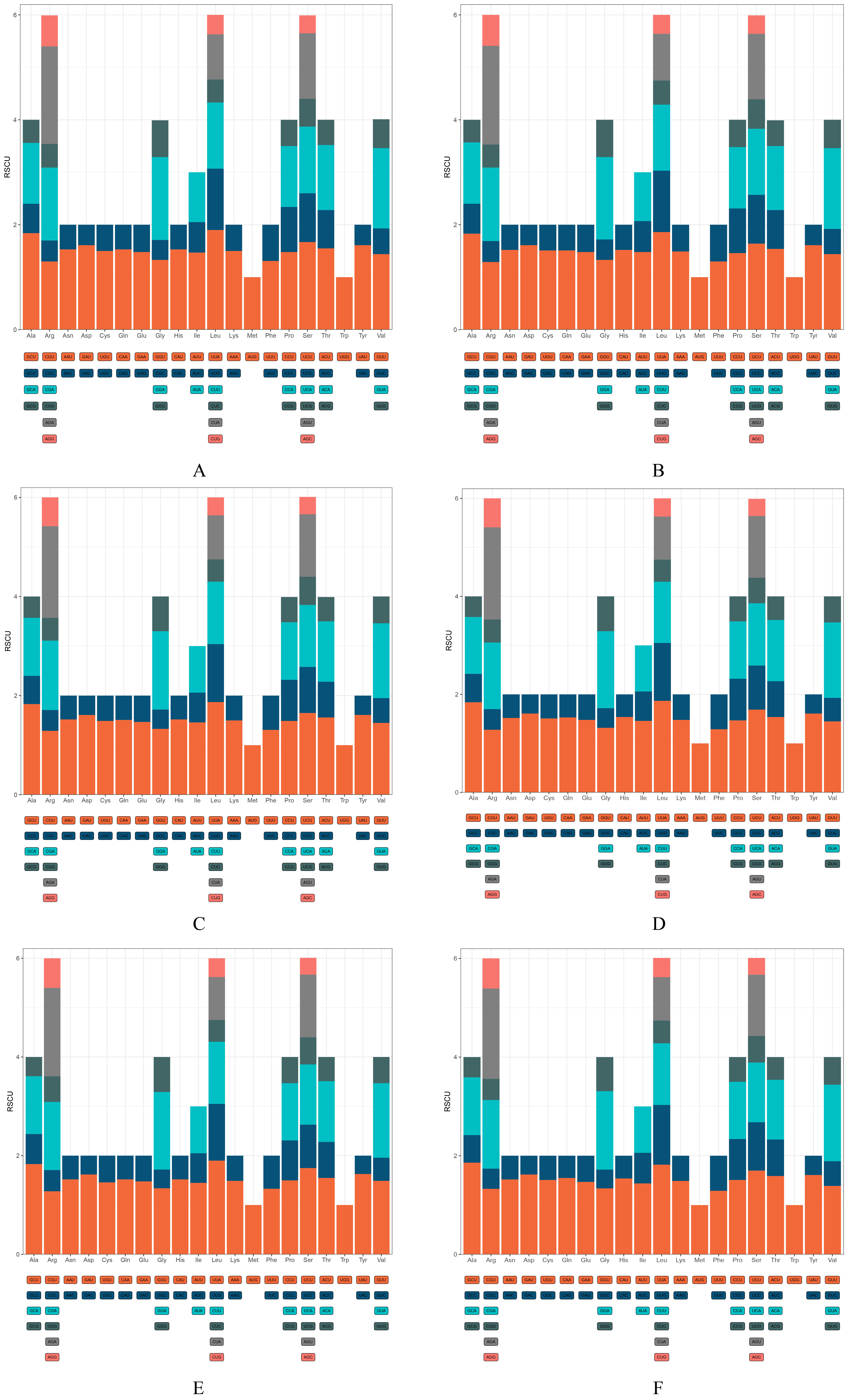

Supplement: Supplementary file 1 [file genes-13-01673-s001.zip › Figure. S1.jpg]

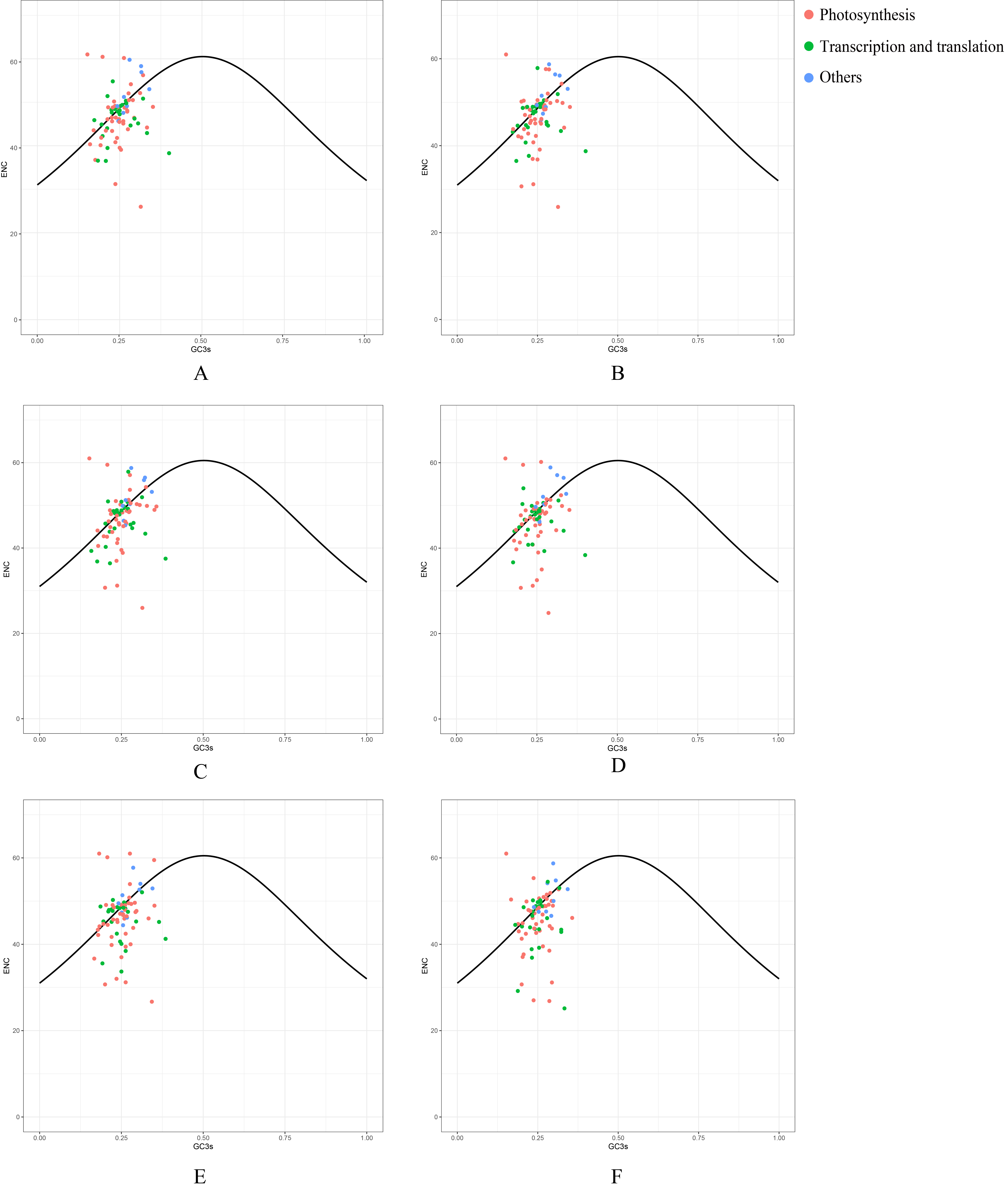

Supplement: Supplementary file 1 [file genes-13-01673-s001.zip › Figure. S2.jpg]

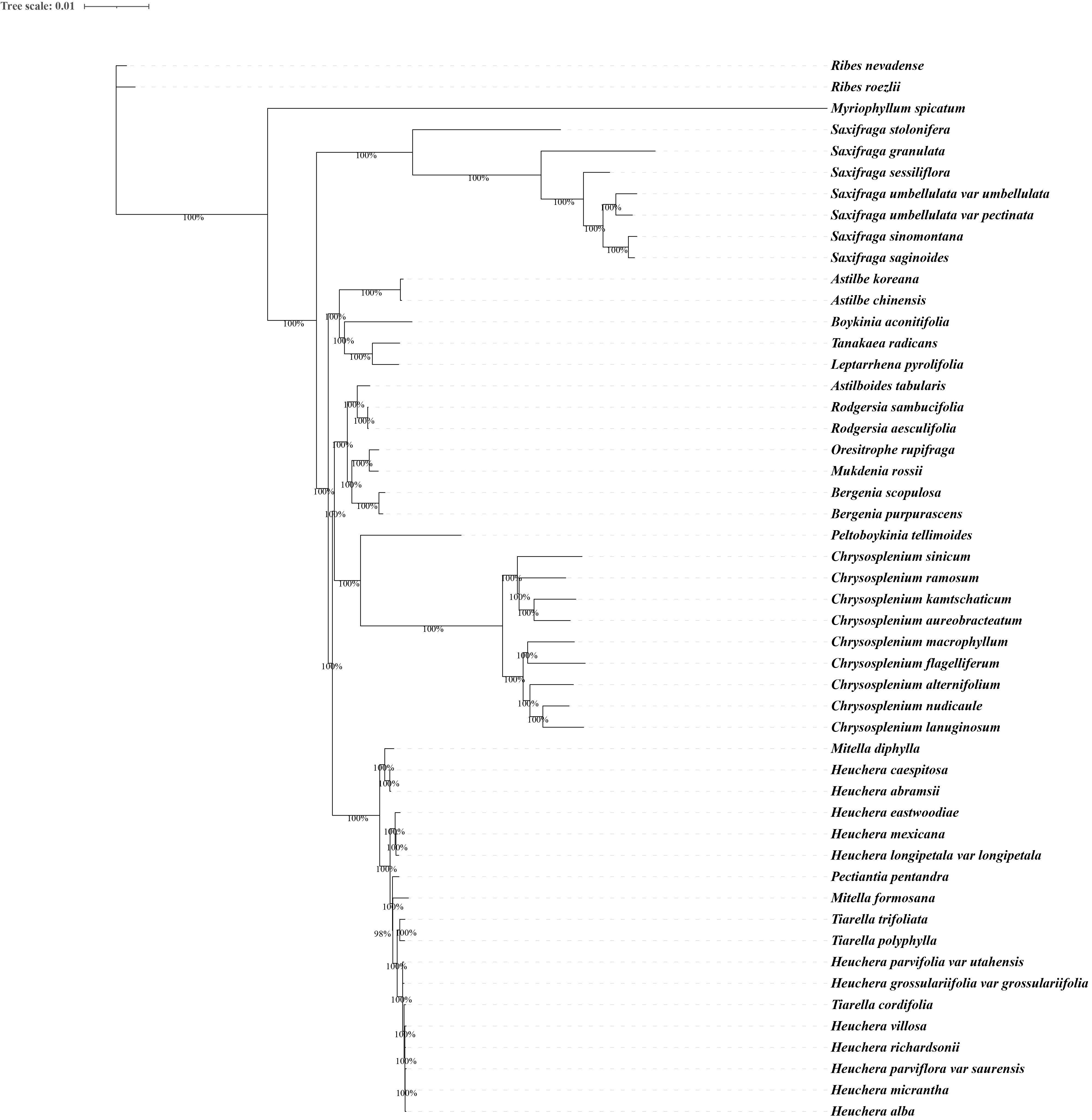

Supplement: Supplementary file 1 [file genes-13-01673-s001.zip › Figure. S3.jpg]
